# A chronic kidney disease patient awareness questionnaire: Development and validation

**DOI:** 10.1371/journal.pone.0216391

**Published:** 2019-05-03

**Authors:** Suyuan Peng, Jiawei He, Jiasheng Huang, Jiaowang Tan, Meifang Liu, Xusheng Liu, Yifan Wu

**Affiliations:** 1 The Second Clinical College of Guangzhou University of Chinese Medicine, Guangzhou, China; 2 Nephrology Department, Guangdong Provincial Hospital of Chinese Medicine, The Second Affiliated Hospital of Guangzhou University of Chinese Medicine, Guangzhou, China; 3 Chronic Disease Management Department, Guangdong Provincial Hospital of Chinese Medicine, The Second Affiliated Hospital of Guangzhou University, Guangzhou, China; University of Mississippi Medical Center, UNITED STATES

## Abstract

**Background:**

With the advance of medical care, chronic non-communicable diseases, like chronic kidney disease (CKD), have become the predominant diseases around the world. With heavy society and economy burden, we shall make full use of chronic disease management, including precision therapies. And the prerequisite for implementing precision medicine is to fully understand the characteristics of patients. Being the basis of the Knowledge-Attitude-Practice Model, patient’s awareness is essential to conduct individualized treatments. However, there have been no validated questionnaires specific to the awareness of patients with CKD. Therefore, this study aims to develop and validate an awareness questionnaire for patients with CKD.

**Methods:**

From March 2013 to September 2014, a cross-sectional study was conducted at Guangdong Provincial Hospital of Chinese Medicine. Age 18 or above were enrolled in the study. After signing the informed consent, they received a self-developed questionnaire to evaluate their CKD-related awareness. Then we collected their demographic data for further analyses. We also conducted item analyses/ validity and reliability analysis to filter out improper items and to retain the eligible ones.

**Results:**

We totally distributed 110 copies of the questionnaires and 100 of them were returned. After item analyses, 2 items were excluded because of Cronbach’s Alpha analysis. In total, 18 items were retained, comprising the final set of the questionnaire. For validity analysis, 4 components could explain the cumulative 73.966% extraction sums of the squared loadings; for reliability analysis, the Guttman Split-Half coefficient was 0.918.

**Conclusions:**

This awareness questionnaire has favorable validity and reliability. It is a sound method for evaluating and measuring levels of disease-related awareness in CKD patients.

## Introduction

In recent decades, we have made great progress in medical care around the world[1]. People lead a better life than ever, with longer life expectancy[2, 3]. However, the epidemiologic trends have changed from acute infectious diseases to non-communicable diseases (NCDs). According to World Health Organization reports, cardiovascular disease, cancer, respiratory diseases, and diabetes are predominant in these NCDs. Besides, chronic kidney disease (CKD) is a common ailment[4]. It’s estimated that nearly 850 million people suffer some form of kidney disease[5]. And the prevalence of CKD worldwide is nearly 11%, which is responsable for nearly 2.4 million deaths[6].

As a lifestyle closely related disease, CKD requires long-term treatments and healthy lifestyle interventions, including diet, medication and exercise[7–11]. In previous diagnosis, and treatment patterns, the evaluation of its curative effect usually focused on the laboratory examinations, which means the changes in disease states. However, several other soft indexes, such as patients’ states of mind and degree of comfort were usually neglected. Thus, in order to implement precision medicine, we need to fully understand their individual characteristics. And the prerequisite is to know their CKD related awareness. In short, only by fully understanding what they don’t know and what they want to know can we carry out targeted treatments.

The Knowledge-Attitude-Practice (KAP) model is a comprehensive procedure covering relevant awareness, attitudes and behavior changes[12]. It was widely used in medical care, education, and management[13–16]. This concept was initially introduced by Schwartz, N.E. in the 1980s[17]. The core of this concept was that once people understand the principles of improving health status, they would change their attitude and behavior. Being as the basis of the KAP Model, patients’ awareness is crucial in clinical practice. We all know that the KAP transition cannot be achieved in the short term, and instead requires the long-term cooperation of patients and healthcare professionals.

Thus, the accurate evaluation of patient awareness is critical. Not only can it evaluate the disease states, but it can also serve as the basis for adjusting intervention measures. However, to date, there is no validated awareness questionnaire for CKD patients. Therefore, it’s an urgent need to develop such a questionnaire, according to the principles and standard processes of questionnaire production[18]. And we hope that this questionnaire is simple, understandable, and representative, which can be applied to the majority of patients with CKD. The present study describes the development and validation of such a questionnaire.

## Methods

### Study design

A cross-sectional study from March 2013 to September 2014 was conducted in Guangdong Provincial Hospital of Chinese Medicine. Then a 20-question questionnaire was randomly distributed to them. Meanwhile, we recorded their demographic data for further analyses. For each question, they were asked to choose only one answer that best suited their actual situation. Patient’s privacy was protected. When necessary, our staff would also have access to their personal data after their enrollment. All process was performed in accordance with the approval of the local Ethics Committee (Ethics Committee of Guangdong Provincial Hospital of Chinese Medicine).

### Patients

We recruited adults aged at least 18 years, who had been diagnosed with CKD according to the 2012 Kidney Disease Improving Global Outcomes practice guidelines. The term “CKD” is defined as kidney damage or glomerular filtration rate <60 mL/min/1.73 m^2^ for 3 months or more, irrespective of cause[19, 20]. For sample size estimation, the common guideline was to recruit four times as many participants as the items’ quantities. Written informed consent was provided by all patient before their enrolment.

### The questionnaire

The entire process involved three procedures—draft, modification, and confirmation stage.

#### Draft stage

By referring to previously published articles and clinical practice, the draft version included four distinct parts.

Part 1 consisted of the first seven questions about the awareness of the disease. This was designed to evaluate their understanding of disease states, prognosis and related prescriptions[20–22]. Through this section, we could realize their general comprehension of the diseases. In clinical practice, we found that some patients just passively received treatments and medical care. It meant that they knew no more than just the name of the disease itself. That was unfavorable to the rehabilitation of the disease.

Part 2 consisted of the next five questions about CKD-related diet and exercise[23–26]. Due to the particularity of their disease, they had been required to focus more on their daily diet and physical activity than healthy ones. Daily consumption of protein, salt, dietary fiber seemed to be correlated with disease progression[27]. Besides, the majority of CKD patients lacked adequate physical activities, which is closely related to adverse outcomes such as glomerular filtration rate decline and dialysis interventions[28]. Unhealthy diet and low level of physical activities were correlated with elevated all-cause risk of death, poorer quality of life and heavier burden. Therefore, we’d better incorporate them into routine clinical practice.

Part 3 was the field of examination results, which was utilized to know patients’ comprehension of laboratory examinations and their connotations. Some indexes, like proteinuria, hematuria and serum creatine, are essential for determining the severity of the disease. Patients needed to check these indicators regularly for more optimized treatments. And they were supposed to know the connotations of their results, rather than passively waiting for the medical staff to inform them. The more active they were, the more likely they would procure better health care. Besides, how to collect the specimen correctly was of importance.

Part 4 covered the awareness of medical resources. This section assessed whether our patients knew how to get in touch with our medical staff in specific situations[29]. In some cases, patients might fail to remain in contact with us when disease progressed. And in this way, they would miss the best opportunity for proper treatment, causing further deterioration of their disease states.

In total, an adapted Likert scale was used for the whole questionnaire. Multiple options included: know nothing about it, know a bit, know basically, know most of it and know clearly. Participants were asked to choose the most suitable answer for each question.

#### Modification stage

The second stage was to modify the questionnaire, and it was as important as the previous stage. Six nephrology experts in our department were invited to propose amendments to the questionnaire. They suggested that the questionnaire needed to highlight the features of renal diseases. Moreover, before the questionnaire could be widely used in clinic, we needed to modify the language readability. Thus, we randomly selected 10 CKD patients with varying educational levels and invited them to complete it. This was done to ensure that most CKD patients could understand all the questions.

#### Confirmation stage

Considering that a proper question should have the features of independence, high-sensitivity, and representativeness, we evaluated all these 20 questions with item analyses so as to screen out any inappropriate ones. After some questions had been thrown out, we conducted construct validity and reliability analyses.

### Evaluation of the questionnaire

#### Item analyses

Item analyses included missing value analysis, small group analysis, item-total correlation analysis, homogeneity analysis, communalities analysis and factor loading analysis.

(1) For missing values analysis, when there were few missing values (commonly no more than 5% of the total number of cases), these values can be presumed to be missing at random[30]. In other words, the majority of CKD patients can understand the questions and make proper choices. The way we deal with these missing values depends on their quantity, type, and reasons[31]. (2)For critical value analysis, it’s used to identify the different extent to the same question by different people. It means that the difference in the average score on the same question between high score group and low score group should be as large as possible. Therefore, this question has better discrimination[32]. (3)For item-total correlation analysis, it’s performed to check if any question is inconsistent with the averaged behavior of the others, and thus can be discarded. If the correlation index between item and total score is too low, it means that they have poor homogenicity[33, 34]. We added all the items and the total score to the variance analysis. Then, we used Pearson correlation coefficient to detect their correlations. (4)For homogeneity, it is the sameness of things. And it is the quality or state of being all the same or all of the same kind. Cronbach Alpha coefficient can be used to detect the internal consistency. Reliability coefficient could be used as one of the indicators for the homogeneity test[35]. Usually, the more questions a questionnaire consists, the larger will the alpha index be. If we delete one of the questions, the alpha index will be lower. But if the index becomes larger, it means that the internal consistency is poor[36]. (5)For communalities, they indicate the amount of variance in each variable that is accounted for. Higher communalities are better. If communalities for a particular variable are low (less than 0.2), then that variable may struggle to load significantly on any factor. In other words, it’s harder for this item to measure scale’s idiosyncracy. (6)For factor loading analysis, it is used to explain the correlations between observed questions using a smaller number of factors[37]. A higher factor value means a closer relationship between the specific question and the whole questionnaire.

#### Factor analysis and validity

A Bartlett test of sphericity and Kaiser-Meyer-Olkin (KMO) measure were then used to test whether we could do the factor analysis[38–40]. Bartlett test of sphericity compares that cocrrelation matrix to the identify matrix. And it means if there is a redundancy between variables. As for KMO analysis, it is a measure of how suitable our date is for the next factor analysis. Its value is between “0” and “1”. If the KMO value is closer to “1”, it indicates that they have more common factors between variables. That is to say, it is more suitable to conduct factor analysis. Then, principal component analysis and varimax rotation were used in the factor analysis.

Validity is the extent to which a conclusion, result and measurement corresponded to the real world. In short, it means the accuracy and correctness. Validity includes internal validity and external validity. Internal validity means the research statements’ accuracy and reality. Besides, the external validity means the inference’s correctness. For the classification of validity, it includes content validity, criterion-lated validity and construct validity. The construct validity is based on the theoretical logic analysis, and at the same time it is based on the actual data obtained to test the correctness of the theory, so it is a rigorous method of validity detection[41].

#### Reliability

The term means the stability and consistency of the results computed by the questionnaire. Results that are highly reliable are reproducible form one study to another. The greater the reliability of the scale, the smaller the standard error it measures. Test-retest reliability, internal consistency, and inter-rater reliability are widely used in different analyses. For the internal consistency, it is the consistency across the items on a multiple-item scale. Split-half correlation can be used to assess the internal consistency[42, 43].

### Statistical analyses

Data analyses were performed using IBM SPSS Statistics version 22.0. All p values less than 0.05 was considered to indicate statistical significance.

Item analyses were conducted by calculating: (1) Missing value analysis. We used 5% as a criterion to delete those items with high omission rates. Then, any missing values were replaced with average values for that item. (2) Critical value analysis. We sorted the items with the highest 27% constituting the high score group and the lowest 27% constituting the low score group. Then, an independent t-test was conducted. If the critical ratio was less than 3.0000, or p-value>0.05, the item was deleted. (3) Item-total correlation. If the correlation coefficient was too low (< 0.4), or if the p-value was insignificant, the item was considered for deletion. (4) Homogeneity. If the Cronbach’s Alpha was higher when the specific item was deleted, then we would discard this item. (5) Communalities and factor loading. If the communalities’ value was less than 0.20, or the factor loading value was less than 0.45, the item could be deleted because of the faint relationship between it and the common factor.

Validity. A Bartlett test of sphericity and a KMO measure were used to test construct validity. We extracted those whose eigenvalues were greater than 0.8.

Reliability. Split-half reliability was used to test the questionnaire’s internal reliability.

## Results

### The questionnaire

Between March 2013 to September 2014, a total of 110 copies of questionnaires were distributed, and 102 of which had returned. Therefore, the recovery rate was 92.73%. Besides, 100 (98.0%) of the returned questionnaires were eligible ones. All CKD patients invited to take the questionnaire were able to complete it within 5 minutes.

The questionnaire contains 18 items, with 7, 5, 4, 2 questions in the awareness of the disease, CKD-related diet and exercise, laboratory examinations and their connotations, and medical resources.

### Demographic characteristic

Overall, mean age was 48.23(SD, 16.37) years. 70 women (70.00%) were enrolled. For kidney function analyses, median serum creatinine was 92.00 (IQR, 65.00, 139.00)μmol/L. The quantity and the proportion of patients with CKD stage 1 to 5 were 32(32.32%), 33 (33.33%), 18(18.18%), 11(11.11%), 5(5.05%) respectively. For the level of education, most people had completed middle school and senior school. And the quantity and proportion for the above education level were 18(36.00%) and 21(42.00%) respectively. For their occupation, 21 people had full-time jobs, accounting for 37.04%. 26 people (48.15%) had retired and 7 people (12.96%) had been looking for a proper job. Besides, only one student (1.85%) was enrolled. (Table 1).

**Table 1:** Demographic characteristic.

Characteristic	n = 100
age, mean (SD), y	48.23 (16.37)
women	70 (70.00%)
serum creatinine, median (IQR), μmol/L	92.00 (65.00, 139.00)
CKD stage	
1	32 (32.32%)
2	33 (33.33%)
3	18 (18.18%)
4	11 (11.11%)
5	5 (5.05%)
education	
primary school	2 (4.00%)
middle school	18 (36.00%)
senior school	21 (42.00%)
associate degree	6 (12.00%)
undergraduate or higher	3 (6.00%)
occupation	
full time	21 (37.04%)
retired	26 (48.15%)
unemployed	7 (12.96%)
student	1 (1.85%)

### Item analyses

(1) For the missing value analysis: 12 items existed missing values. Besides, all the omission rates were less than 5%, between 1% to 3%. (2) For the critical value analysis, all the critical ratios were higher than 3.0, with all p-values less than 0.05. (3) Item-total correlation. All the correlation coefficients were above 0.4, with all the p-values less than 0.05. (4) Homogeneity. The Cronbach’s Alpha coefficient was 0.946, but if item No. 1 or No. 6 was deleted, the coefficient would increase to 0.947. (5) Communalities and factor loading. The communality values for No. 1 and No. 6 were 0.253 and 0.261, respectively. Moreover, their factor loadings were all greater than 0.45. In short, items No. 1 and No. 6 were deleted. The rest items were all reserved for further analyses. Other data not mentioned were all represented in Table 2.

**Table 2:** Item analyses.

				homogeneity			suggestion
Item	omission rate (%)	Critical Ratio	Item-Total Correlation	Cronbach’s Alpha if Item Deleted	Commu-nalities	Factor loading	Reserved (R) or Deleted (D)
No.1	.0	5.563	.522	.947	.253	.503	D
No.2	2.0	7.386	.618	.945	.377	.614	R
No.3	3.0	11.889	.762	.943	.587	.766	R
No.4	3.0	9.680	.766	.943	.596	.772	R
No.5	2.0	8.927	.671	.944	.435	.660	R
No.6	3.0	5.504	.543	.947	.261	.511	D
No.7	2.0	7.407	.639	.945	.413	.643	R
No.8	.0	7.590	.639	.945	.419	.647	R
No.9	.0	8.616	.784	.942	.610	.781	R
No.10	1.0	10.514	.800	.942	.663	.814	R
No.11	2.0	10.366	.733	.943	.544	.737	R
No.12	.0	11.229	.777	.942	.628	.792	R
No.13	1.0	10.799	.702	.944	.478	.691	R
No.14	.0	12.554	.790	.942	.629	.793	R
No.15	.0	10.054	.761	.943	.590	.768	R
No.16	1.0	10.341	.723	.943	.535	.731	R
No.17	.0	9.411	.755	.943	.585	.765	R
No.18	2.0	11.179	.819	.942	.692	.832	R
No.19	1.0	9.734	.731	.943	.526	.725	R
No.20	.0	7.649	.645	.945	.398	.631	R
criterion	≤5.0	≥3.000	≥.400	≤0.946	≥.200	≥.450	

### Construct validity

For the Bartlett test of sphericity, χ2 was 1,286.017, and the p-value was 0.000 <0.005. Also, the KMO measure was 0.91, which was higher than 0.8. We extracted 4 components, which had an explanatory power of 73.966%. Component 1 consisted of No. 5 and Nos. 10–14. Component 2 consisted of Nos. 7–8, and Nos. 15–18. Component 3 consisted of Nos. 2–4 and No. 9. Component 4 consisted of Nos. 19–20 (Tables 3–5).

**Table 3:** Kaiser-Meyer-Olkin and Bartlett’s test of sphericity.

Kaiser-Meyer-Olkin Measure of Sampling Adequacy.		.910
Bartlett’s Test of Sphericity	Approx. Chi-Square	1286.017
	df	153
	Sig.	.000

**Table 4:** Construct validity.

	Component				Communalities
	1	2	3	4	
NO.13	.828	.232	.119	.101	.764
NO.14	.772	.228	.169	.390	.829
NO.11	.743	.329	.186	.149	.716
NO.12	.649	.286	.345	.298	.710
NO.5	.613	.121	.383	.126	.553
NO.1	.606	.347	.428	.219	.718
NO.17	.204	.757	.232	.351	.792
NO.16	.280	.757	.096	.343	.778
NO.15	.336	.718	.111	.371	.778
NO.7	.254	.668	.345	-.046	.631
NO.8	.204	.617	.512	-.088	.693
NO.18	.328	.502	.495	.365	.738
NO.2	.165	.163	.788	.100	.684
NO.3	.252	.324	.676	.295	.712
NO.9	.350	.217	.625	.369	.696
NO.4	.327	.188	.579	.504	.732
NO.20	.170	.192	.184	.829	.786
NO.19	.405	.230	.253	.614	.658

Extraction Method: Principal Component Analysis.

Rotation Method: Varimax with Kaiser Normalization.

Rotation converged in 7 iterations.

**Table 5:** Total variance explained.

Component	Initial Eigenvalues			Extraction Sums of Squared Loadings		
	Total	% of Variance	Cumulative %	Total	% of Variance	Cumulative %
1	9.865	54.808	54.808	9.865	54.808	54.808
2	1.378	7.655	62.463	1.378	7.655	62.463
3	1.048	5.825	68.287	1.048	5.825	68.287
4	1.022	5.679	73.966	1.022	5.679	73.966
5	.699	3.883	77.849			
6	.684	3.803	81.652			
7	.625	3.473	85.125			
8	.462	2.569	87.694			
9	.396	2.198	89.891			
10	.328	1.823	91.714			
11	.280	1.557	93.271			
12	.249	1.382	94.653			
13	.231	1.283	95.937			
14	.192	1.065	97.001			
15	.170	.944	97.946			
16	.141	.785	98.730			
17	.120	.664	99.395			
18	.109	.605	100.000			

Extraction Method: Principal Component Analysis.

### Reliability

The correlation between the forms was 0.852. The Spearman-Brown length-related coefficient was 0.920, and the Guttman Split-Half coefficient was 0.918 (Table 6).

**Table 6:** Reliability.

Cronbach’s Alpha	Part 1	Value	.898
		N of Items	9^a^
	Part 2	Value	.915
		N of Items	9^b^
	Total N of Items		18
Correlation Between Forms			.852
Spearman-Brown Coefficient	Equal Length		.920
	Unequal Length		.920 .918
Guttman Split-Half Coefficient			

^a^The items are: NO.2, NO.3, NO.4, NO.5, NO.7, NO.8, NO.9, NO.10, NO.11.

^b^The items are: NO.12, NO.13, NO.14, NO.15, NO.16, NO.17, NO.18, NO.19, NO.20.

## Discussion

CKD has become the predominant diseases around the world, with a heavy social burden. Various kinds of risk factors attribute to the initiation and exacerbation of the diseases. Unhealthy lifestyles or diets, inappropriate physical activities, incorrect use of medicines may affect kidney functions and damage the structure. In order to conduct precision therapies, understanding patients’ awareness seems to be significant. And this way, we can understand what they lack or what they want to know. It sounds easy to make it happen. However, in clinical practice, it’s hard to achieve this goal, especially in developing countries or undeveloped countries. By referring to relevant guidelines and articles, we hope to develop an awareness questionnaire, which can be used widely among CKD patients. That is our original intention to do this research.

Through the standardized process mentioned above, we developed a CKD related awareness questionnaire, which can be used to know patient awareness of the disease, diet and exercise, laboratory results, and medical resources. We used perspicuous statements to constitute our questionnaire. We did so because we hoped this version of questionnaire could be understanded by the majority of CKD patients. Vague words or specialized vocabulary were avoided if possible. And in our hospital, a Chinese version of questionnaire was used instead. From our demographic analysis, various kinds of subgroup CKD patients were enrolled, which had certain representativeness. And in reality, all the enrolled patients could finish the whole questionnaire in 5 minutes. Maybe, this questionnaire could be widely used among CKD patients. (Table 7).

**Table 7:** Questionnaire.

	know nothingabout it	know a bit	know basically	know most of it	know clearly
1.Do you know what symptoms will develop when you get worse?					
2.Do you know what aggravates your kidney function?					
3.Do you know the long-term prognosis of your disease?					
4.Do you know how to control your blood pressure?					
5.Do you know the names and usage of your medicines?					
6.Do you know the primary role of your medicines?					
7.Do you know which medicine may impair the kidney function?					
8.Do you know what are unhealthy diets?					
9.Do you know what contains high-quality protein?					
10.Do you know food which should be avoided?					
11.Do you know how much salt to be used daily?					
12.Do you know what exercise fits you?					
13.Do you know what laboratory examinations you should regularly check?					
14.Do you know how to collect your urine correctly?					
15.Do you know the meaning of your test reports?					
16.Do you know how to evaluate your curative effect?					
17.Do you know what kind of educational activities are organized regularly in our clinic?					
18.Do you know how to contact our medical staffs when you have a question?					

In the recruitment stage, we enrolled those diagnosed with CKD, irrespective of cause. One common guideline was to enroll 4 times patients as the items quantities. However, to develop a more accurate questionnaire for CKD patients, we would need to expand our sample size, ideally by a factor of at least 10. Only with such a sizable sample could we reduce the effects of research bias for variables such as age, sex, and education level.

For statistical analyses, item analyses were used to filter those improper items. Firstly, all the items’ omission rates were less than 5%, which meant all the questions were easily understood. Since it was displayed in the form of Likert scale, it was much easier to make the best choice. For critical value analysis, highest score group and lowest score group had nice discrimination. All critical ratios met the established criterion. In the study, we used the former 27% and the latter 27% to consist of the highest score group and lowest score group. This method and theory derived from questionnaire identification analysis method. In the conventional model reference test, if the test scores were normally distributed, the authenticity for the discrimination would be maximized. For item-total correlation analysis, all the correlation coefficients were above 0.4. If the coefficients were less than 0.4, it meant that the specific item is inconsistent with the averaged behavior of the others. Therefore, we could discard this item in practice. Moreover, in homogeneity analyses, we adopted Cronbach’s Alpha, communalities and component matrix analysis. They measured the differences of similarities between several items. If the Cronbach’s Alpha was higher when the specific item was deleted, or communalities values were less than 0.2, or the factor loading values were less than 0.45, they all meant that the homogeneity was weak. And we could consider deleting them.

After the item analyses, we kept the rest of 18 items to test their construct validity and reliability. For the validity and reliability analysis, this questionnaire showed nice results. They indicated that this questionnaire had fine construct validity, stability, and consistency.

The whole process included the development and statistical analyses were mentioned above. Through this questionnaire, we could understand what patients lack and what they wanted to learn. So, we can better educate them for targeted treatments.

However, there were some disadvantages in this study. First of all, it was the sample size estimation. Currently, we had no uniform standards in the field of questionnaire. Methods, objectives, requirements and data could determine the size of the sample size. We had referred to some relevant studies and taken actual situation into consideration. This questionnaire consisted 20 items initially, so 80 participants might be enough. But the larger the sample size, the more representative the sample was of the population. We thought we might need more participants in the future, like 200 or 300. Moreover, more demographics details should be recorded for better representativeness. The cause of CKD was very complicated, and we needed to record more details, like their lifestyle and family history, medication history, surgical history, allergy history, and complications.

We hope this questionnaire could play a significant role in the evaluation of patient awareness in chronic disease management. Only through the joint efforts of both doctors and patients, can chronic diseases can be controlled to the greatest possible extent.

## Conclusion

After statistical analyses, this final questionnaire had nice validity and reliability. Therefore, it is our hope that it will help to understand patients’ awareness of their renal disease.
